# Retraction: Hypoxia-Inducible MiR-210 Is an Independent Prognostic Factor and Contributes to Metastasis in Colorectal Cancer

**DOI:** 10.1371/journal.pone.0244280

**Published:** 2020-12-17

**Authors:** The PLOS ONE Editors

Following the publication of this article [1], concerns were raised regarding the results presented in Figs 4 and 5, as well as similarities between panels presented in this article and an article published the following year in *PLOS ONE* [2].

Specifically,

- The following panels appear similar:
  ○ The SW480 miR-210 mimics panel of Fig 4A [1], the Migration NC panel of Fig 4C [2], and the Migration NC panel of Fig 4D [2].
  ○ The SW480 miR-210 inhibitor panel of Fig 4B [1], the Migration miR-230 mimics panel of Fig 4C [2], and the Migration miR-203 inhibitor panel of Fig 4D [2].
  ○ The SW480 NC panel of Fig 4C [1] and the Invasion miR203 mimics panel of Fig 5C [2].
  ○ The SW480 NC panel of 4D [1] and the Invasion siRNA ZNF217 panel of Fig 5C [2].
  ○ The SW480 miR-210 inhibitor panel of Fig 4D [1], the Invasion miR-230 mimics panel of Fig 4C [2], and the Invasion miR-203 inhibitor panel of Fig 4D [2].
  ○ The Migration Hypoxia-NC panel of Fig 5B [1] and the Migration siRNA ZNF217 panel of Fig 5C [2].
  ○ The Migration Hypoxia-mimics panel of Fig 5B [1] and the Invasion siRNA NC panel of Fig 5C [2].
  ○ The Migration Normoxia-NC panel of Fig 5D [1] and the Migration miR203 mimics panel of Fig 5C [2].
- Partial overlap has been detected between the following panels:
  ○ The Migration Hypoxia-inhibitor panel of Fig 5D [1] and the Invasion ZNF217 siRNA panel of Fig 2C [2].
  ○ The Invasion Normoxia-NC panel of Fig 5A [1] and the Invasion Hypoxia-inhibitor panel of Fig 5C [1].
  ○ The SW480 NC panel of Fig 4D [1] and the Invasion siRNA ZNF217 panel of Fig 5C [2] partially overlap with the following panels:
    ■ SW480 NC of Fig 4C [1].
    ■ SW480 miR-210 mimics panel of Fig 4C [1].
    ■ Invasion Normoxia-NC of Fig 5B [1].
    ■ Invasion Hypoxia-inhibitor of Fig 5D [1].
    ■ Invasion NC of Fig 4C [2].
    ■ Invasion NC of Fig 4D [2].
    ■ Invasion miR203 mimics panel of Fig 5C [2].
  ○ The Migration Hypoxia-NC panel of Fig 5B [1] and the Migration siRNA ZNF217 panel of Fig 5C [2] partially overlap with the Invasion Hypoxia-inhibitor panel of Fig 5D [1].
  ○ The Invasion Normoxia-NC panel of Fig 5B [1], the Invasion Normoxia-NC panel of Fig 5D [1], and the Invasion Hypoxia-inhibitor panel of Fig 5D [1].

The authors stated that the similarity observed between the Invasion SW480 NC panels is due to these panels representing the same experimental conditions. In addition, the authors stated that the similarity between the panels in [1] and [2] are the results of errors in the preparation of the figures presented in [2], and that the similarity between the remaining panels listed above are the result of inadvertent errors introduced during figure preparation for [1]. The authors provided data underlying their published results, however, the extent of the image concerns observed in this article raise serious concerns regarding the handling of the data obtained during this study and the integrity of the published results. In light of the concerns affecting multiple figure panels that question the integrity of these data, the *PLOS ONE* Editors retract this article.

XZ and AQ agreed with the retraction. LD, YY, HL, JL, LW, YL, ZD, XJ, HW, ZL, and GZ either did not respond directly or could not be reached. CW did not agree with the retraction.

## References

[1] Qu A, Du L, Yang Y, Liu H, Li J, Wang L, et al (2014) Hypoxia-Inducible MiR-210 Is an Independent Prognostic Factor and Contributes to Metastasis in Colorectal Cancer. PLoS ONE 9(3): e90952 10.1371/journal.pone.0090952 24632577 PMC3954583

[2] Li Z, Du L, Dong Z, Yang Y, Zhang X, Wang L, et al (2015) MiR-203 Suppresses ZNF217 Upregulation in Colorectal Cancer and Its Oncogenicity. PLoS ONE 10(1): e0116170 10.1371/journal.pone.0116170 25621839 PMC4306553

